# Reframing Our Health Care System for Patients With Hearing Loss

**DOI:** 10.1044/2022_JSLHR-22-00052

**Published:** 2022-08-15

**Authors:** Michael McKee, Tyler G. James, Kaila V. T. Helm, Brianna Marzolf, Dana H. Chung, John Williams, Philip Zazove

**Affiliations:** a Department of Family Medicine, University of Michigan/Michigan Medicine, Ann Arbor; b Department of Population Health Science, University of Mississippi Medical Center, Jackson

## Abstract

**Purpose:**

Nearly 20% of U.S. Americans report a hearing loss, yet our current health care system is poorly designed and equipped to effectively care for these individuals. Individuals with hearing loss report communication breakdowns, inaccessible health information, reduced awareness and training by health care providers, and decreased satisfaction while struggling with inadequate health literacy. These all contribute to health inequities and increased health care expenditures and inefficiencies. It is time to reframe the health care system for these individuals using existing models of best practices and accessibility to mitigate inequities and improve quality of care.

**Method:**

A review of system-, clinic-, provider-, and patient-level barriers, along with existing and suggested efforts to improve care for individuals with hearing loss, are presented.

**Results:**

These strategies include improving screening and identification of hearing loss, adopting universal design and inclusion principles, implementing effective communication approaches, leveraging assistive technologies and training, and diversifying a team to better care for patients with hearing loss. Patients should also be encouraged to seek social support and resources from hearing loss organizations while leveraging technologies to help facilitate communication.

**Conclusions:**

The strategies described introduce actionable steps that can be made at the system, clinic, provider, and patient levels. With implementation of these steps, significant progress can be made to more proactively meet the needs of patients with hearing loss.

**Presentation Video:**

https://doi.org/10.23641/asha.21215843

The adverse effects of hearing loss are profound and varied, affecting one's ability to perform independent activities of daily living, as well as their quality of life (Gopinath et al., 2012; Shukla et al., 2020) and overall health. Individuals with hearing loss experience social isolation, lower income (Agrawal et al., 2008; Blanchfield et al., 2001), poorer physical and psychological health (McKee et al., 2018; Solheim et al., 2011), increased risk of falls and hospitalization (Danermark & Gellerstedt, 2004; Genther et al., 2015; Lin et al., 2011), and greater rates of cognitive decline than their peers with normal hearing (Livingston et al., 2017). Patients with hearing loss also utilize health care more frequently, yet the health care they receive is often ill designed to appropriately meet their needs (Reed, Altan, et al., 2019). This places these individuals at elevated risk for adverse health events and lower adherence (Mick et al., 2014). Communication breakdowns (Mick et al., 2014), inaccessible health information, and inadequate health literacy impact the ability of patients with hearing loss to successfully navigate health care (Chang et al., 2018; McKee, Moreland, et al., 2015; McKee, Paasche-Orlow, et al., 2015). Despite these identified challenges, there remains a lack of awareness and training by health care providers on how to identify, manage, and care for those with a hearing loss (Hoang et al., 2011; Zazove et al., 2017). Despite a relatively high hearing loss prevalence, affecting at least 17% of all adults in the United States and over 50% among those over 80 years of age (Lin et al., 2011), few health care programs or training resources exist to improve health care for individuals with hearing loss.

It is critical to examine how health care systems can become more effective and inclusive in their care for individuals with hearing loss. Our objectives for this review article are to identify key health care barriers that exacerbate disparities for individuals with hearing loss and outline ways to reframe the delivery of health care to promote a system that is both equitable and accessible. A review of the literature, along with best practices and models around the care for individuals with hearing loss, follows. For ease of understanding, the article has been divided into three major sections: clinic- and system-, provider-, and patient-level issues and approaches (see Figure 1). Brief sections on COVID-19 challenges and strategies along with how hearing health professionals can promote equitable health care for individuals with hearing loss are also shared.

**Figure 1.**
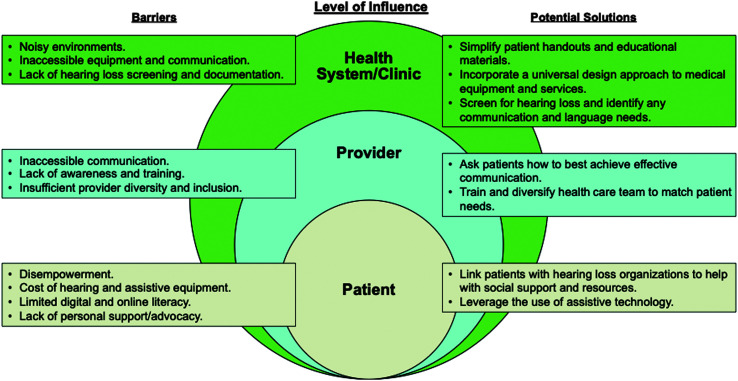
Health system-, provider-, and patient-level opportunities and strategies to address the needs of patients with hearing loss.

## Clinic- and System-Level Issues and Approaches

Hearing loss, despite being at least partially addressable by the use of listening assistive devices or through the provision of effective accommodations, is largely overlooked by health care providers. On a per-capita basis, individuals with hearing loss incur more health care expenditures than those without hearing loss ($22,000 over 10 years), even after adjusting for sociodemographic and health factors (Reed, Altan, et al., 2019). Emergency department utilization is higher among individuals with hearing loss, ranging from 16.9% among those with untreated hearing loss (Reed, Altan, et al., 2019) to nearly double among those who use American Sign Language (ASL; James et al., 2021; McKee, Winters, et al., 2015). Patients with untreated hearing loss are also at higher risk of longer hospital stays (Mitra et al., 2020; Reed, Altan, et al., 2019). Individuals with mild and moderate severity hearing loss have 16% and 21% higher hospitalization risk, respectively, than their hearing peers (Genther et al., 2015). Hearing loss, especially if it is untreated or unaddressed, significantly impacts health care utilization and outcomes. Yet, few systems, clinicians, or hospitals routinely screen for hearing loss to recognize those at risk for communication breakdowns. This makes it difficult to identify accommodations or communication assistance that a patient with a hearing loss may benefit from (Bushman et al., 2012; Morris et al., 2021). The lack of widespread screening also adversely affects the referral rates to those who can address hearing loss (e.g., hearing aids; Bushman et al., 2012; DeJonckheere et al., 2021; Zazove et al., 2020). Because of this, 75% of hearing loss remains undiagnosed and untreated (Zazove et al., 2017).

Health care environments are noisy, with daytime and nighttime noise ranging from 37–88 dB to 38.7–68.8 dB, respectively (de Lima Andrade et al., 2021), negatively impacting patient–provider communication and patient well-being. This poses significant obstacles for people with hearing loss, making it challenging to understand nurse call buttons or TVs without closed captioning. The use of masks by health care teams—universal during the COVID-19 pandemic—adds additional hurdles to effective communication (Homans & Vroegop, 2021).

Many of these issues are not only solvable but are required to be addressed by the Americans with Disabilities Act (ADA; United States Department of Justice, Civil Rights Division, 2013) and Patient Protection and Affordable Care Act (ACA; United States Department of Health and Human Services, 2010). These legal mandates regulate health care communication access, including the provision of accommodations that are necessary to achieve effective communication for individuals with hearing loss. Titles II and III of the ADA prohibit discrimination at any health care facilities (United States Department of Justice, Civil Rights Division, 2005, 2013). The addition of the ACA's Section 1557 further strengthened the requirement that health care sites respect communication and accommodation requests by patients with hearing loss (United States Department of Health and Human Services, 2010). This can range from auxiliary aids (e.g., pocket talkers) to services (e.g., sign language interpreters). To effectively meet the needs of patients with hearing loss and comply with ADA's and ACA's mandates, several best practices already exist to reduce these barriers (see Table 1). These strategies should be included in mandatory education around health care communication frequently offered to health care providers and staff at health care systems.

**Table 1.** Actionable steps for health clinics/systems.

Recommendation	Implementation
Standardize screening for hearing loss and any related communication and language needs.	Display hearing loss related communication needs in a visible area of the patients' chart Create alerts to call American Sign Language (ASL) interpreters if applicable
Incorporate a universal design approach to medical equipment and services to reduce the need for last minute adaptation or inability to provide accommodation for patients with specific needs.	Use sound and visual alerts (e.g., flashing light door alerts to notify patients when someone knocks on the door) Provide hearing and communication assistive devices (e.g., pocket talkers, Video Remote Interpreting)
Simplify patient handouts and educational materials using health literacy principles.	Use plain language Incorporate pictures/graphics Optimize layouts and guides Provide patient educational videos with captions and availability in ASL

**Figure 2.**
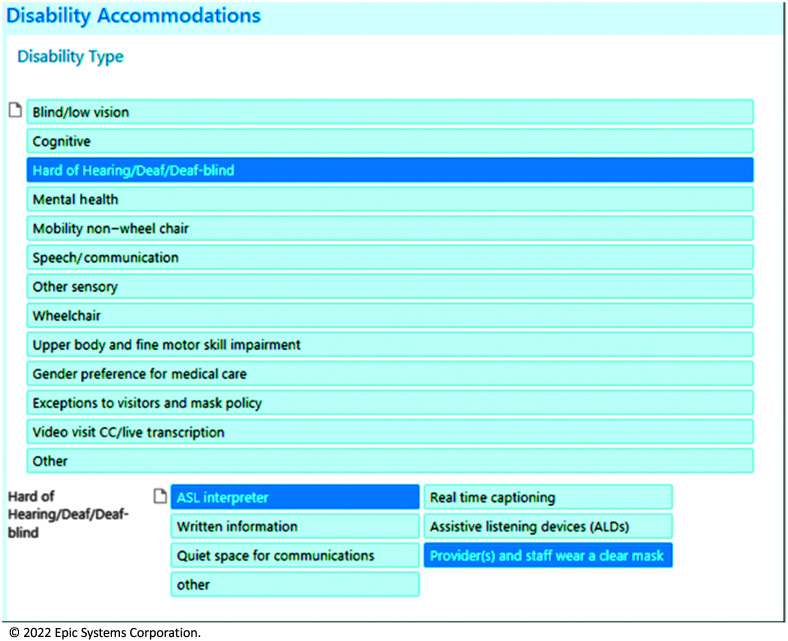
Screenshot of Michigan Medicine's “Disability and Accommodations” tab. ASL = American Sign Language. Reprinted with permission of Epic Systems Corporation.

First, health care systems should standardize screening for hearing loss and any related communication and language needs. This presence of hearing loss from this screening can then be displayed in patients' storyboards in the electronic health records, available for all health care team members to view. This protocol has been implemented in some health care systems, including the University of Colorado Health and University of Michigan/Michigan Medicine, and found not to be bothersome to patients (Morris et al., 2021). Collecting information about hearing loss and communication needs upfront enables health care systems to be more proactive in arranging necessary accommodations or communication requests. At Michigan Medicine, this questionnaire is either available on patient facing portals for patients to manage, or it can be directly entered by health care team members based on patient-provided information. For example, Michigan Medicine's “Disability and Accommodations Tab” (Center for Disability Health and Wellness, 2022) is located in the patient's storyboard of the electronic health record and includes information about patient disabilities and requested accommodations (see Figure 2). Different levels of integration between these disability tabs are available. For instance, health systems can create alerts to call ASL interpreters should patients indicate that they communicate in ASL. However, since some health care systems do not provide ASL as a language option within the electronic health record, informatics teams in these systems must work to include ASL as a language option to improve delivery of care to these patients (James et al., 2021).

Second, incorporating a universal design approach to medical equipment and services reduces the need for last minute adaptation or accommodation for patients with specific needs. Most equipment alarms, bed alerts, or overhead announcements are auditory based (Jones, 2021; United States Department of Justice, Civil Rights Division, 2005; Mestayer, 2019); health care settings should encourage devices that offer both sound and visual alerts—similar to fire alarm requirements—and tactile alerts when available. Visual alerts could be easily integrated into many health care situations. Patient rooms, for instance, could have flashing light door alerts to notify patients when someone knocks on the door. These devices can be portable or hard-wired and would help allay anxieties (e.g., if undressing or in bathroom) or stress (being startled) that may diminish communication quality when the health care team member arrives. Part of the Hearing Loss Association of America's (n.d.) communication access plan suggested the need for a vibrating pager while in the waiting room. Furthermore, TVs or tablets in patient or waiting rooms should have captioning as a default, as all patients can benefit from captioning due to background noises or information processing disabilities.

Clinics and hospital units (emergency room and inpatient) should have hearing and communication-assistive devices available upon patient requests, including personal amplifiers (e.g., pocket-talkers) that can improve communication access for patients with hearing loss. Pocket-talkers help reduce background noise while amplifying the speaker's voice sent directly to the connected ear phones worn by the patient with a hearing loss. These relatively inexpensive tools are useful for patients with milder forms of unaided hearing loss. A recent pilot study demonstrated the feasibility of providing personal amplifiers for patients with a hearing loss in a hospital setting and documented the users' improvement in quality and satisfaction of health care communication with inpatient nurses (Kimball et al., 2018). Other accommodation devices include the availability of web- or iPad-based captioning or interpreting services (i.e., Video Remote Interpreting), which patients may only prefer at certain times (James et al., 2022), and Communication Access Realtime Translation (CART) as a form of real-time captioning.

Patient facing platforms offer additional opportunities to improve information access in the clinic and at the bedside. Some examples include programs such as MyChart Bedside, a service offered through Epic, that provides patients access to their chart, recent diagnoses, and lab results, and opportunities to contact care team members while hospitalized (Epic, 2019). These services facilitate communication between patients and their care team, enhance patients' understanding of their health care conditions, and improve the patient experience (Winstanley et al., 2017). Triage areas, including checking in and out areas, can also include templates or printouts of talking points (e.g., laminated cards) to facilitate accessible communication (National Library of Medicine, 2021; Panzer et al., 2020).

Third, simplify the above-mentioned patient handouts and educational materials using health literacy principles. Health literacy is defined as the ability of an individual to obtain, process, and understand basic health information needed to make appropriate health decisions (National Library of Medicine, 2021). Health information is often complex and can benefit from steps such as using plain language written preferably at a sixth grade or lower reading levels, pictures and/or graphics, optimized layouts and guides (e.g., bullets or arrows), and captioned videos (Jacobson & Parker, 2014; Zazove et al., 2013). Improving accessibility is a critical first step in addressing health literacy issues for those with hearing loss. Patients with hearing loss frequently have inadequate health literacy (McKee, Paasche-Orlow, et al., 2015; Wells et al., 2020), likely due to inaccessible information and communication. Presenting information at an accessible literacy level helps the large number of patients with low literacy, not just those with hearing loss. However, the use of plain language or health literacy tools does not equate accessibility. For example, patient educational videos may be well designed and effective for most patients, but if they are not captioned and available in ASL (Hickey et al., 2013), they continue to be inaccessible for many patients with more severe hearing loss. Accessibility and inclusion-based features are a precursor to comprehension. Therefore, all materials shared through patients' smart TVs, tablets, and patient portals should be accurately captioned. This includes public service announcements and critical health information, which should also be available in ASL.

## Provider-Level Issues and Approaches

Health care providers, including their support staff, are rarely trained to effectively care for and communicate with individuals with hearing loss. Health care training programs and medical schools allot little or no time for disability-based topics, including hearing loss (Agaronnik et al., 2019). As a result, providers are poor judges of patients' communication needs (Kruse et al., 2021); thus, communication is often ineffective. One study found that 87% of providers felt they communicated effectively with their Deaf signing patients, even in the absence of interpreters, through lipreading and note writing (Alexander et al., 2012). Yet, patients with hearing loss commonly state they have problems communicating with their physicians (Schneider et al., 2010; Wallhagen, 2010). Among 20 physicians that were interviewed in a study exploring how they primarily communicated with Deaf patients, 13 reported that they had access to ASL interpreters, and 10 had TeleTYpe/Telecommunications Device for the Deaf (teletypewriters) at their clinic. Still, many of the physicians testified that writing notes, lipreading, talking more slowly, or even shouting into the good ear of the patient were the primary strategies used, despite their limited effectiveness in delivering critical medical information (Agaronnik et al., 2019).

Most health care providers believe that using sign language interpreters to communicate with Deaf signing patients is preferable, but few use interpreters in their practices (Alexander et al., 2012; Ebert & Heckerling, 1995). In a 2018 survey of Deaf people in Florida, James et al. (2021) found that 37% of people were denied an interpreter in a health care facility in the previous year. When asked about their responsibilities with the ADA, 35.8% of 714 physicians surveyed knew little or nothing about their legal obligations and 71.2% answered incorrectly regarding who determines reasonable accommodations (Iezzoni et al., 2022). The lack of training and of providing accommodations among health care providers has been confirmed in other studies (Barnett, 2002; Ebert & Heckerling, 1995; James et al., 2022; McKee, Moreland, et al., 2015). This failure to accommodate patients with hearing loss impacts their quality of health care while placing health care providers at litigation risk. Several steps can help health care providers ensure that communication is both effective and accessible (see Table 2).

**Table 2.** Actionable steps for health care providers.

Recommendation	Implementation
Routinely screen for hearing loss	Ask patients, “Do you think you have hearing loss?” Refer to audiology for further testing if necessary
Ask patients with hearing loss how to best achieve effective communication	Assess patient communication needs and document in chart
Document preferred communication strategy in the medical record	Update patients storyboard in the electronic medical record, if available. Alternatively, document preferred communication strategy in note or add to problem list Ensure documentation is clear and accessible to other members of the care team
Make appropriate accommodations, including ASL interpreters, communication access real time translation (CART), or other aids including personal sound amplification products	Be aware of legal mandates around effective communication Implement clinic based protocols to ensure accommodations are provided upon request
Optimize patient rooming environment	Minimize background noise Exam rooms should be well lit, without any visual obstacles (e.g., laptop) obstructing the providers face or lips
Directly engage patient	Speak clearly, at a normal pace Wear a clear or transparent face mask to facilitate lipreading and recognition of facial cues
Check patient comprehension	Use teach back or teach to goal method Simplify patient educational materials using health literacy principles

First, include routine hearing loss screening in health care. Most hearing loss remains underdiagnosed by primary care providers (Wallhagen, 2010), since many do not suspect or consider hearing loss unimportant (Zazove et al., 2017; Zazove et al., 2020). Screening for hearing loss at a health care provider level is both feasible and effective. Zazove et al. (2020) demonstrated that a single question, “Do you think you have a hearing loss?” prompted by an electronic alert significantly improved identifying people at risk for hearing loss. It also increased documentation in the medical record and referrals to audiology for further testing and, if applicable, hearing aid fitting. Health care providers should recognize the importance of screening for hearing loss, not just to recognize when communication breakdowns are likely to occur, but to improve management and reduce the sequalae of hearing loss (e.g., decreased cognitive function or worsening mental health). Hearing loss screeners may remind health care providers to discuss how hearing loss may be affecting a patient's communication and quality of life (DeJonckheere et al., 2021). This can help prioritize the management of hearing loss and address ongoing hearing loss stigma in health care and even among patients themselves (Wallhagen, 2010). Similarly, other hearing loss screeners such as the Hearing Handicap Inventory for the Elderly–Screening have proven beneficial in detecting hearing loss among older adults in health care settings (Feltner et al., 2021).

Second, ask patients with hearing loss how to best achieve effective communication. Health care providers should defer to patients with hearing loss, whose life experiences can help determine optimal communication strategies. Shared communication strategies should be highlighted in the patient's record to help proactively prepare for upcoming health care visits and notify other team members about patient communication preferences. Most patients with hearing loss can benefit from several basic communication approaches. The patient rooming environment should be well lit and without any visual obstacles, to maximize patient viewing of a provider's face and lips (e.g., computer between provider or patient or nontransparent masks). Laptops or computers used for medical record access or documentation should be positioned to the side, or providers should consider using a medical scribe to help with record keeping. The direct engagement of the patient with a hearing loss helps foster trust and improve the quality of communication (Mick et al., 2014). Health care providers should speak clearly and at a normal pace. Any background noise should be minimized to help patients with hearing loss understand what is being said. Avoidance of window glare due to the provider standing before a window is important. Clear or transparent masks approved by the U.S. Food and Drug Administration (FDA) should be incorporated during any interactions with patients with hearing loss (see COVID-19 Challenges and Approaches section below for more information). This helps facilitate lipreading or recognition of facial cues necessary for improving communication between a provider and a patient. Since hearing loss is recognized as a major source of communication breakdowns, it is valuable to check patient comprehension (McKee, Moreland, et al., 2015). The use of teach back (Agency for Healthcare Research and Quality, 2015) or teach to goal (Baker et al., 2011) to ensure patients understand what is being communicated has been found to be effective. Teach back helps to check patient's comprehension by asking these individuals to state in their own words what they need to know or do about their health (“show me” method; Agency for Healthcare Research and Quality, 2015). Teach to goal takes this a step further to have the patient demonstrate ability to comfort and self-care (e.g., ability to adjust settings on a hearing aid).

Moreover, health care providers need to be aware of their legal responsibilities and provide accommodations when requested by patients (Agaronnik et al., 2019). Accommodation costs should not be viewed in the context of a single health care visit but from the perspective of an overhead business expense of running a health care facility. Accommodations can include services such as qualified sign language interpreters, CART, or other aids such as personal sound amplification products (Agaronnik et al., 2019).

Third, expand training opportunities for health care providers on how to care for patients with hearing loss. The omission of formal disability health education in medical school has led the U.S. Surgeon General (Office of the Surgeon General & Office on Disability, 2005) and the World Health Organization (2011) to call for strengthening education regarding care for patients with disabilities, including those with hearing loss. Many barriers in health care stem from health care providers' lack of community-specific cultural and linguistic competency when working with members of underserved groups (Hoang et al., 2011). A review of the literature reveals surprisingly few studies that describe effective training to improve communication with individuals with hearing loss. The ones identified included the use of modules (Deuster et al., 2010; Kruse et al., 2021), standardized patients (Lowenstein et al., 2009), and immersion programs (Hoang et al., 2011), and were small-scale, one-time projects with a limited number of participants. This pales in comparison with studies around improving communication quality among other populations, including ethnic minority groups (Govere & Govere, 2016; Renzaho et al., 2013), patients with cancer (Bos–van den Hoek et al., 2019), patients with dementia (Eggenberger et al., 2013), and in end-of-life care (Lord et al., 2016). One particularly promising program for individuals with hearing loss, Deaf Strong Hospital, provides a half day immersion program for first year medical students to improve their understanding on how communication barriers affect one's ability to navigate and receive health care through a role-reversal exercise (Mathews et al., 2011; Thew et al., 2012). These medical students become “patients” of Deaf community members who serve as “doctors.” Programs like this provide a vivid opportunity for trainees to learn often overlooked barriers. The program's success has allowed for the expansion to other training programs (Thew et al., 2012), including pharmacy (Mathews et al., 2011). Programs like these should be universally included in medical school curriculum. Humanities and sign language courses also offer indirect opportunities for students to learn more about the health care needs of individuals with hearing loss, including Deaf signers (Farber et al., 2004; Panzer, 2022). Hearing health professionals, with their training and recognition of the effects hearing loss has on their patients, should be incorporated into the curriculum of other non–hearing health professional training programs as well as continuing medical education programs. This interdisciplinary training can help improve the implementation of hearing screenings, testing, and treatment in a culturally effective manner.

Fourth, diversify the health care team to better care for a patient population with diverse needs. Training a diverse health care workforce provides opportunities to improve care for a larger segment of the population. When the health care workforce mirrors the background of patients, it leads to positive effects on patient care and access for marginalized groups (Cooper-Patrick et al., 1999; McKee et al., 2013; Moreland et al., 2013; Takeshita et al., 2020). For example, Moreland et al. found that deaf and hard of hearing physicians are more likely to serve patients with hearing loss (Moreland et al., 2013). Prior research suggests that increasing racial and ethnic concordance between patients and physicians can significantly enhance cross-cultural communication and patients' participation in clinical decision making, intentions to adhere to clinicians' recommendations, and satisfaction with health care experience (Cooper-Patrick et al., 1999). This finding is expected to be the same for those with hearing loss, especially those with shared life experiences (McKee et al., 2013).

Health care members with disabilities, including hearing loss, bring unique insights to the medical and audiology field as a result of their personal experience and knowledge. This insight can directly benefit both patients and colleagues by providing patient-centered care with greater empathy (Spillman, 2021), addressing attitudinal barriers to care, and creating a more culturally competent workforce. In a study conducted by Meeks and Jain (2018), out of 6,000 surveyed physicians, only 22 (0.4%) reported having a hearing disability. Moreland et al. (2013) showed that physicians with hearing loss are more likely to care for people with hearing loss. It is unknown how many hearing health professionals, including audiologists, have a hearing loss (Nouri et al., 2021).

Removing application barriers at health professional schools and training programs, such as restrictive technical standards, can help improve the recruitment and retention of those with disabilities and hearing loss into health care and audiology fields (Meeks & Jain, 2018; Zazove et al., 2016). Restrictive technical standards, unlike functional technical standards, requires that the student be able to display physical, cognitive, and sensory abilities (e.g., “*student* must be able to hear *heart sounds*”; McKee et al., 2016; Stauffer et al., 2022). A student with a hearing loss would normally be discouraged from applying even though existing accommodations and assistive technologies can overcome limitations caused by the hearing loss. In contrast, a functional technical standard allows students with a hearing loss to incorporate assistive technologies and accommodations to successfully complete the essential tasks in their training and work (McKee et al., 2016; Stauffer et al., 2022).

## Patient-Level Issues and Approaches

For many, hearing loss disrupts various facets of their lives, including how they access and use health care. Hearing loss can be isolating, resulting in a loss of social support and resources to address their needs (Shukla et al., 2020). Hearing aids, a potential tool to address hearing loss, remain largely not covered by most insurance plans and beyond reach for many from an affordability standpoint (Jilla et al., 2020). Patient satisfaction with health care is decreased among those with a hearing loss (Reed, Bertz, et al., 2019), resulting in a sense of disempowerment that may encourage avoidance of health care, including preventive care services and outpatient services (Barnett & Franks, 2002; McKee et al., 2011). Individuals with hearing loss may have limited knowledge of their legal rights to accessible health care. This can lower their ability to self-advocate or seek strategies to address health care-based discrimination or inaccessibility (Steinberg et al., 2006). To address these issues, one should consider several key strategies to empower patients with hearing loss (see Table 3).

**Table 3.** Actionable steps for patients.

Recommendation	Implementation
Encourage networking with hearing loss organizations to help with patient empowerment	List hearing loss organizations and helpful resources designed for patients and consumers with hearing loss
Leverage the use of assistive technology to address some of their communication needs	Display key assistive technologies that are adoptable by those with hearing loss Inform upcoming technology changes, including those by the Over-the-Counter Hearing Aid Act

First, encourage patients with hearing loss to seek social support and resources from hearing loss organizations such as the Hearing Loss Association of America (2018) or the National Association of the Deaf (2022). These organizations provide advocacy services, resources, and networking opportunities relevant to hearing loss. Many of their efforts center around advocating for better care and accessibility in health care. These organizations also regularly disseminate news briefs, blogs and vlogs on topics such as legislation (e.g., insurance coverage for hearing aids), communication tips, and tools (e.g., communication or language apps) for both patients and health care providers. One strategy to empower patients with hearing loss is to encourage these individuals to fill out and complete a communication access plan (Hearing Loss Association of America, n.d.) prior to hospitalizations or surgeries. This plan replicates many of the similarities to birth plans that many parents do prior to the arrival of their child. This plan, along with patient-based guides (Hearing Loss Association of America, 2018), are aimed to improve the communication accessibility and quality at upcoming health care visits, proactively arrange accommodations, and prepare these individuals for any items that may be needed (e.g., back up hearing aid batteries or battery chargers). Additionally, hearing loss organizations' lobbying efforts were instrumental to the passage of the Over-the-Counter Hearing Aid Act that will help many consumers with mild to moderate hearing loss to afford cheaper hearing aids (many likely from smartphone manufacturers) without a prescription (Food and Drug Administration, 2021). This is anticipated to not only improve hearing aid access but also lower costs especially among those who lack hearing health insurance coverage.

Second, leverage the use of assistive technology to address some of their communication needs. Patients now have more technological options to consider than ever before to help with communication (Zitelli & Mormer, 2020). Smartphones can access a variety of automated captioning apps for a nominal cost. These apps provide another in-person communication tool when utilized either on the patient's personal or even clinic-provided devices—if the patient desires. These apps involve speaking slowly and clearly into the device's microphone so that one's voice can be transcribed for the listener. Apps are usually computer generated and based on artificial intelligence. Therefore, they are not perfect and often have higher rates of errors with background noises or accents. Thus, speaking slowly and distinctly at a comfortable volume is important for better transcription. There are also apps that utilize live streaming operators who listen remotely and type what is said, these are more accurate but costlier. Fortunately, automatic speech recognition (ASR) platforms, incorporating machine learning, are improving rapidly and will soon offer a suitable alternative for personal transcription needs. Some existing ASR platforms including Google Live Transcribe, Otter.ai, and Interact Streamer are widely available and affordable for many to use. It is anticipated that the use of signing avatars and greater use of wearables with built-in communication tools will soon be available to use in a variety of settings (Quandt, 2020).

As more app-, Internet-, and other electronic-based tools become available and integrated into daily communication approaches, health care providers and health care systems need to recognize digital literacy gaps—gaps common among older and lower income adults, particularly those with a hearing loss (Levy et al., 2015). Additionally, these exciting new technologies do not replace the need for increased training and cultural/linguistic competencies for health care providers. While these options are available for patients, the onus is not on the patient but on health care providers and systems to provide this access in health care environments.

## COVID-19 Challenges and Approaches

The COVID-19 pandemic greatly impacted health care communication and accessibility for individuals with hearing loss (McKee et al., 2020; Moreland et al., 2021; Wilson et al., 2021). Health care systems widely mandated personal protective equipment, including surgical or protective masks, to ensure the safety of workers and patients. As a result of this change, some accommodations, including interpreters, were temporarily unavailable in person. The quick shift to virtual visits was often done without any accompanying accessibility features (McKee et al., 2020; Moreland et al., 2021). Even though the legal mandates, such as the ADA and ACA, remain in place, these changes exacerbated many health care inequities for individuals with hearing loss, especially since many were adopted and implemented without the input of patients with hearing loss. To address some of the health care barriers that were created by the pandemic, health care systems have implemented several steps. These changes should be implemented nationwide to remain in compliance with the ADA. First, virtual visits need to ensure that captioning is available, either by inserting a remote captionist or using a live transcription (AI-based) with some telehealth platforms (e.g., Zoom). Under the ACA, health care organizations should defer to the patient's preference on the specific type of captioning service. Moreover, multi- or three-way video visits allow for the integration of ASL interpreters to facilitate health communication between providers and patients with hearing loss. Clear masks are now available for health care workers to use that allow individuals with hearing loss to see faces/lips, including two FDA-approved masks Safe N′ Clear Communicator and ClearMask. Clear or transparent masks have been demonstrated to improve communication quality, trust, and recognition of expressions between providers and patients (Chu et al., 2021; Kratzke et al., 2021; Thibodeau et al., 2021). These masks can be universally adopted since they benefit everyone, not just those with hearing loss.

## Ways Hearing Health Professionals Can Promote Greater Health Care Equity for Individuals With Hearing Loss

Hearing health professionals play an integral role in helping individuals with hearing loss better manage their health. This can include identification and management of hearing loss, screening for associated health conditions (e.g., dementia) and providing advocacy and guidance on assistive devices and services that are rarely addressed by other health care providers. Integrated care models offer an avenue for hearing health professionals to collaborate more closely with primary care providers (D'Onofrio & Zeng, 2022; Weinstein, 2015). This model of care has been used widely in behavioral health and for the management of other types of disabilities, notably intellectual and developmental disabilities and Deaf signers (Ervin et al., 2014; Panzer et al., 2020; Reiter et al., 2018). Due to the high prevalence of hearing loss among older adults, this model is feasible among clinics who care for a large proportion of geriatric patients. Furthermore, psychologists, social worker, or other behavioral health specialists can be trained to address unique needs of patients with hearing loss experiencing mental health conditions. This may include tailored counseling services that are focused on ideal coping strategies and self-advocacy training to help maintain social engagement and reduce functional limitations secondary to hearing loss.

Hearing health professionals' training and awareness of hearing loss' effects could position these professionals to be more effectively involved in chronic disease management and surveillances. This may include cognitive decline, fall prevention, and adherence to different medication regimens. Likewise, hearing health professionals need to directly engage and educate other health professionals on the importance of screening and addressing hearing loss.

The ACA Section 4302 outlines the importance of improving health data collection and the ability to analyze the sources of health disparities. Although it has not been widely implemented, this section contains provisions to strengthen health care data collection efforts for primary language (e.g., ASL) and disability (e.g., hearing loss; Office of the Assistant Secretary for Planning and Evaluation, 2011). Hearing health professionals must push for the inclusion of hearing health and other disability-related questions in health care data collection systems and surveillances. The inclusion of this information will improve our understanding of health care disparities and identify optimal intervention targets for patients with hearing loss.

Additional research is needed to determine how to improve the low screening and audiology referral rates by primary care providers. Hearing aids, cochlear implants, assistive hearing devices, and coping strategies have been demonstrated to preserve the patients' ability to maintain their social activities and communication access yet usage rates among patients with hearing loss remain poor (Chien & Lin, 2012; Reed et al., 2021). Ownership of these devices remains problematic due to high costs, lack of insurance coverage, vanity, stigma, and hearing health access issues (McKee et al., 2019). The Over-the-Counter Hearing Aid Act seeks to address some of these barriers, yet concerns remain. Hearing health professionals need to be wary of exacerbating existing hearing health disparities. Older adults are not only more likely to struggle with inadequate health literacy but also with lower digital literacy. This will place these individuals at greater risk of falling further behind, with resulting impacts on health and health care use. Hearing health professionals will need to work closely with manufacturers to ensure that instructions for over-the-counter hearing aids and devices are clearly described, including how and when to see hearing aid specialists. Access to these specialists will require an expansion of tele-audiology and a push for new reimbursement models (D'Onofrio & Zeng, 2022).

## Conclusions

Hearing loss globally affects health and health care. It is not only common but remains largely overlooked, resulting in significant inequities and adverse outcomes for patients with hearing loss. Reframing health care to proactively meet the needs of patients with hearing loss is both attainable and necessary. The strategies described throughout the article introduce actionable steps that can be made at the system, clinic, provider, and patient levels. This will require the efforts of hearing health professionals, organizations, and patient advocates to ensure that it becomes a priority of our health care system.

## Supplementary Material

10.1044/2022_JSLHR-22-00052SMS1 Presentation Video Presentation Video
